# Hepatitis B virus X protein suppresses caveolin-1 expression in hepatocellular carcinoma by regulating DNA methylation

**DOI:** 10.1186/1471-2407-12-353

**Published:** 2012-08-15

**Authors:** Jun Yan, Qian Lu, Jiahong Dong, Xiaowu Li, Kuansheng Ma, Lei Cai

**Affiliations:** 1 Institute of Hepatobiliary Surgery, Southwest Hospital, Third Military Medical University, Chongqing, 400038, China; 2 Department of Hepatobiliary Surgery, PLA General Hospital, Beijing, 100853, China

**Keywords:** Hepatitis B virus X protein, Hepatocellular carcinoma, Caveolin-1, Methylation

## Abstract

**Background:**

To understand the molecular mechanisms of caveolin-1 downregulation by hepatitis B virus X protein (HBx).

**Methods:**

The DNA methylation status of the caveolin-1 promoter was examined by nested methylation-specific PCR of 33 hepatitis B virus (HBV)-infected hepatocellular carcinoma (HCC) samples. The SMMC-7721 hepatoma cell line was transfected with a recombinant HBx adenoviral vector, and the effects of HBx protein on caveolin-1 expression and promoter methylation were examined and confirmed by sequencing. A reporter gene containing the caveolin-1 promoter region was constructed, and the effects of HBx on the transcriptional activity of the promoter were also studied.

**Results:**

Methylation of the caveolin-1 promoter was detected in 84.8% (28/33) of HBV-infected HCC samples. Expression of caveolin-1 was significantly downregulated (P = 0.022), and multiple CpG sites in the promoter region of caveolin-1 were methylated in SMMC-7721 cells after HBx transfection. Transfected HBx significantly suppressed caveolin-1 promoter activity (P = 0.001).

**Conclusions:**

HBx protein induces methylation of the caveolin-1 promoter region and suppresses its expression.

## Background

Hepatitis B virus (HBV) is one of the leading causes of hepatocellular carcinoma (HCC). Among the four open reading frames in the HBV genome (S, C, P and X), the HBx protein is the most implicated in the pathogenesis of HCC. HBx protein is involved in multiple steps of carcinoma development and activates several signal transduction pathways that lead to transcriptional upregulation of a number of genes including growth factor genes and oncogenes. In addition, HBx promotes cell cycle progression, inactivates negative growth regulators, such as p53, and facilitates tumor invasion and metastasis [1-3].

Caveolin-1 is a candidate tumor suppressor gene [4,5]. We previously reported that caveolin-1 expression is significantly decreased in HBV-infected HCC tissues and closely correlates with tumor progression. Although a negative correlation between caveolin-1 and HBx expression was also found, it is unclear whether HBx leads to caveolin-1 downregulation [6]. Because hypermethylation in promoter regions of tumor suppressor genes is often induced by viral infection, and inactivation of tumor suppressors is a major cause of carcinogenesis, we hypothesized that HBx might regulate caveolin-1 expression by a similar mechanism.

## Methods

### Materials

The SMMC-7221 hepatoma cell line was purchased from the Cell Bank of the Chinese Academy of Sciences (Shanghai, China) and confirmed as not infected with HBV.

Thirty-three HCC tissue samples were obtained from 29 male and 4 female patients between 2006 and 2007 at the Institute of Hepatobiliary Surgery, Southwest Hospital (Chongqing, China). The average age of patients was 42.3±10.6. Patients did not receive radiotherapy or chemotherapy before surgical removal of the affected liver. All patients had chronic HBV infection, and HCC was confirmed by pathological studies. Healthy liver tissues were obtained from donated livers during liver transplantation surgery. This study met the requirements of the Declaration of Helsinki, and was approved by the Research Ethics Committee of the Southwest Hospital. Informed consent was obtained from all participants.

### Immunohistochemistry

Immunohistochemical staining was performed on 5 μm tissue sections using a rabbit anti-human caveolin-1 polyclonal antibody (Santa Cruz Biotechnology, Japan). For caveolin immunostaining, sections were treated with 3% H_2_O_2_ for 15 minutes, and then antigen retrieval was performed by boiling in 0.01 M citrate buffer in a microwave for 12 minutes. Sections were then immersed in phosphate-buffered saline for 20 minutes, incubated for 20 minutes in a milk-peroxide blocking solution, and then incubated with the primary antibody (1:500 dilution) overnight at 4°C. Incubated with the secondary antibody (goat anti-rabbit antibody, Zhongshan Golden Bridge Biotechnology, China) for 2 hours, and development with diaminobenzidine Sections were counterstained with hematoxylin.

### Cell culture and transient transfection

SMMC-7721 hepatoma cells were maintained in Dulbecco’s modified eagle medium (DMEM) supplemented with 10% fetal bovine serum (FBS) (Hyclone, USA) at 37°C with 5% CO_2_. Recombinant adenoviral vectors containing either the HBx gene (HBx-ayw subtype adenoviral vector [7]; the original plasmid was kindly provided by Dr. David Chan, University of Hong Kong) or control green fluorescent portein (GFP) gene were used to infect 80% confluent SMMC-7721 cells. To reduce the cytotoxicity of adenovirus infection, the culture medium was changed after 6–8 hours. GFP expression was confirmed by fluorescence microscopy after 48 hours post-infection, and the transfection efficiency was approximately 80%. Cells were then harvested for the following experiments.

### Real-time PCR

Total RNA from SMMC-7721 hepatoma cells was extracted by Trizol (Invitrogen, USA), and cDNA was synthesized by reverse transcriptase (Cat#: DRR03s; Takara, Dalian, China) according to the manufacturer’s instructions. Caveolin-1 and HBx expression were examined by RT-PCR kits (DRR041s; Takara) using a thermocycler (LightCycler; Roche, Switzerland). Primer sequences were as follows: caveolin-1, forward 5′-AGA CGA GCT GAG CGA GAA-3′ and reverse 5′-GCA GAC AGC AAG CGG TAA-3′; HBx, forward 5′-GCA CCT CTC TTT ACG CGG ACT-3′ and reverse 5′-TAC AAA GAC CTT TAA TCT AAT C-3′; β-actin, forward 5′-CGG GAA ATC GTG CGT GAC-3′ and reverse 5′-TGG AAG GTG GAC AGC GAG G-3′. cDNA from MHCC-97 L hepatoma cells was used as a control for caveolin-1 analysis. cDNA from HepG2 hepatoma cells transiently transfected with HBx was used as a control for HBx analysis. Real-time PCR products were also examined by gel electrophoresis.

### Western blotting

SMMC-7721 cell lysates were prepared at 72 hours post-transfection and quantified by the Bradford method. Protein samples were examined by 10% SDS-PAGE analysis, transferred to PVDF membranes, blocked with 5% milk and incubated with a primary mouse anti-HBx monoclonal antibody (1:500 dilution)or a rabbit anti-human caveolin-1 polyclonal antibody (1:500 dilution) at 4°C overnight. Samples were then incubated with a secondary goat anti-mouse or goat anti-rabbit IgG H + L antibody at 37°C for 2 hours. Blots were developed by a chemiluminescence kit (Pierce, USA). β-actin was used as an internal reference.

### Nested methylation-specific PCR (n-MSP)

**Figure 1:**
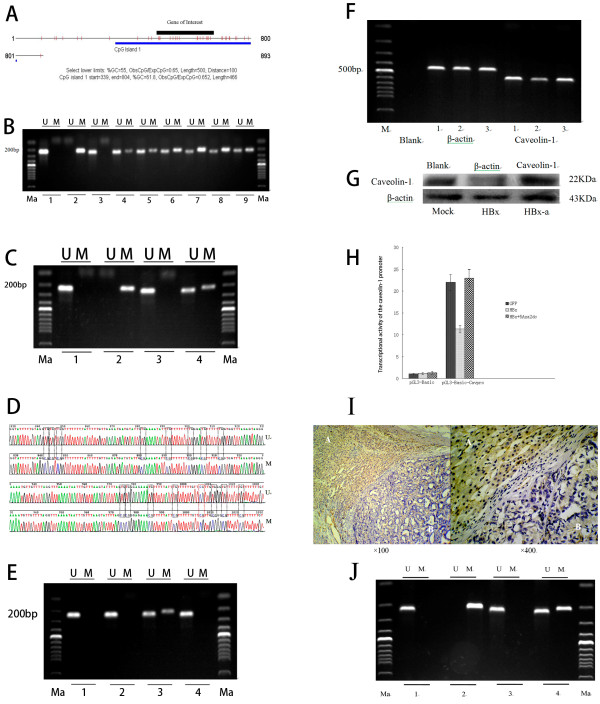
**HBx induces DNA methylation in the promoter region of the caveolin-1 gene.** (**A**) There is a CpG island in the promoter region of the caveolin-1 gene. Gene of Interest: The targeted gene that we studied. (**B**) DNA methylation status in HCC tissues was examined by n-MSP. U: unmethylated PCR products, M: methylated PCR products. 1: healthy liver tissue, 2: M.Sss I-treated healthy liver tissue, 3–9: HCC liver tissues. (**C**) DNA methylation status in SMMC-7721 cells was examined. 1: healthy liver tissue, 2: M.Sss I-treated healthy liver tissue, 3: mock-treated cells, 4: HBx-transfected cells. (**D**) Methylated CpG sites in the caveolin-1 promoter region were identified by sequencing. Black boxes represent newly methylated CpG sites after HBx transfection. Red boxes represent de novo methylated CpG sites. U: Pmd-18 T-Cav-U, M: Pmd-18 T-Cav-M. (**E**) DNA methylation status was examined in SMMC-7721 cells, 1: mock treatment, 2: (Mock-a) mock and 5′-Aza-2′-DC treatment, 3: HBx transfection, 4: (HBx-a) HBx transfection and 5′-Aza-2′-DC treatment. (**F**) Caveolin-1 mRNA was examined at 48 hours post-infection by RT-PCR, 1: mock treatment, 2: HBx transfection, 3: HBx transfection and 5′-Aza-2′-DC treatment. (**G**) Caveolin-1 protein expression in SMMC-7721 cells was examined at 48 hours post-infection by western blot. Mock: SMMC-7721 cells were transfected with an empty GFP recombinant adenoviral vector. HBx: HBx transfection, HBx-a: HBx transfection and 5′-Aza-2′-DC treatment. (**H**) Transcriptional activity of the caveolin-1 promoter was determined by a dual luciferase reporter assay. pGL3-Basic: empty vector, pGL3-Basic-Cavpro: recombinant pGL3-Basic containing the caveolin-1 promoter. (**I**) Expression of caveolin-1 in HCC and adjacent tissue with HBV infection, as determined by immunohistochemistry. A: adjacent non-tumor tissue, B: HCC. (**J**) DNA methylation status in adjacent non-tumor tissue and HCC was examined by n-MSP. U: unmethylated PCR products, M: methylated PCR products. 1: healthy liver tissue, 2: M.Sss I-treated healthy liver tissue, 3: adjacent non-tumor tissue, 4: HCC.

DNA methylation usually occurs in CpG islands in promoter regions. We analyzed the promoter sequence of the caveolin-1 gene (NCBI ID: AF019742) to determine whether it contained CpG islands (http://ccnt.hsc.usc.edu/cpgislands2/cpg.aspx). A typical CpG island sequence was found in this region as shown in Figure 1A. Caveolin-1 MSP-specific PCR primers were designed as shown in [8,9]: outside forward 5′-AGGATAGGGTAGGATTGTGG-3′ and outside reverse 5′-CATAAA ACATTCCTAACTTCTCTTCACCTC-3′; methylated forward 5′-GGTATTTTTGTAGGCGCGTC-3′ and methylated reverse 5′-CTAACAACAAAAAACGAAAAACG-3′; unmethylated forward 5′-GTTTATATTGGGTATTTTTGTAGGTGTGT-3′ and unmethylated reverse 5′-TCCCCAAAATTCTAACAACAAAAAACAAAAAAC-3′. The location of the DNA fragments is shown in Figure 1A. DNA from SMMC-7721 cells, healthy liver tissue, and HCC liver tissue was extracted and treated with a DNA methylation conversion kit (Zymo Research, Beijing). Nested PCR was performed first with outside primers and a high GC-content PCR kit (LA PCR Kit, Takara). First PCR products were diluted 30-fold before being used as a secondary PCR template. Then, methylation-specific and non-methylation PCR were performed. PCR products were separated using gel electrophoresis. Healthy liver tissue DNA was used as an unmethylated positive control and a methylated negative control. Tissue DNA treated with CpG methylase (M.Sss I; NEB, USA) was used as a methylated positive control and an unmethylated negative control.

### Sequencing

To characterize CpG methylation sites in the promoter region of the caveolin-1 gene, PCR products from control SMMC-7721 and HBx-transfected SMMC-7721 cells were ligated into a Pmd18-T plasmid (Takara) and designated as Pmd-18 T-Cav-U and Pmd-18 T-Cav-M, respectively. DNA sequences were analyzed by Invitrogen (Shanghai, China).

### 5′-Aza-2′-DC treatment

SMMC-7721 hepatoma cells were seeded in 6-well plates and 5′-Aza-2′-DC (Sigma, USA) was added to wells. DNA, RNA, and protein were extracted after 72 hours to perform n-MSP, RT-PCR and western blot analyses.

### Plasmid construction

The caveolin-1 promoter region (AF019742.1, NCBI GenBank) was amplified from healthy liver tissue DNA. The primers used were: forward primer, 5′-CGGGGTACCCTTTCCTCACAGCCAAGCACAT-3′ and reverse primer, 5′-CCGCTCGAGTTTGGAGAGGCAGATAGCAGAAG-3′. PCR products were separated by gel electrophoresis, extracted and inserted into a Pmd-18-T plasmid. DH10B competent cells were transformed with recombinant plasmids. Transformed bacteria were cultured, and plasmids were extracted using E.Z.N.A Plasmid Miniprep Kit (Omega Bio-tek US), Then, plasmids were cleaved by Kpn and Xho restriction endonucleases to confirm the insert. Finally, DNA fragments were inserted into a pGL3-Basic plasmid (Promega, US) and confirmed by restriction enzyme digestion and sequencing.

### Dual-Luciferase reporter assay

A reporter plasmid containing the caveolin-1 promoter region (pGL3-Basic-Cavpro) was amplified, extracted and purified. A pRL-TK vector (Promega, USA) was used as a control. SMMC-7721 cells were seeded in 96-well plates. After 24 hours, the luciferase transfection complex and recombinant adenoviral vector were added to the culture medium. Some wells were also treated with 5′-Aza-2′-DC. Culture medium was changed after 8 hours, and luciferase activity was assessed after 48 hours using pGL3-Basic as a control.

### Statistical analysis

Statistical analysis was performed using SPSS 13.0 software. A *T*-test was used for comparison between two groups, and one-way analysis of variance was used for comparison between more than two groups. A value of P < 0.05 was considered statistically significant.

## Results

### HBx downregulates caveolin-1 expression in SMMC-7721 cells

**Figure 2:**
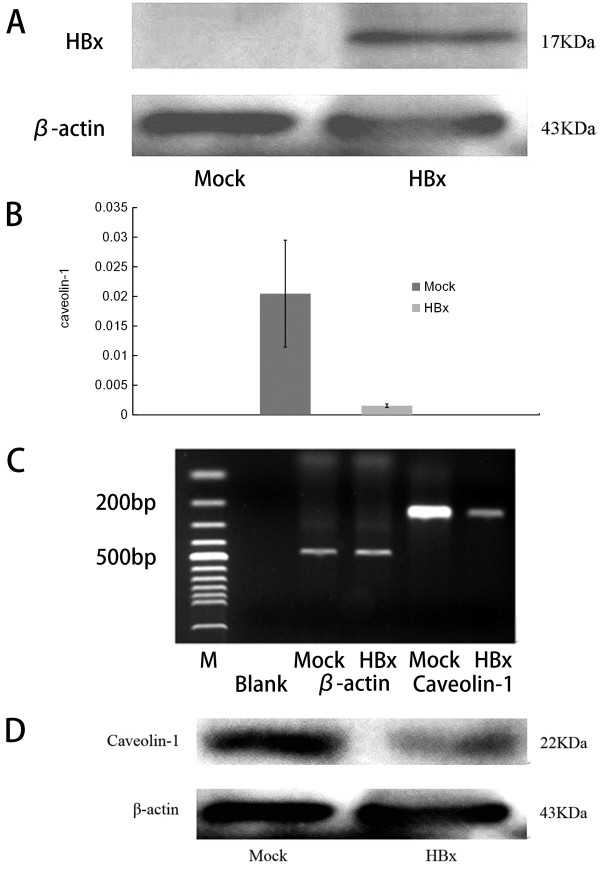
**HBx downregulates caveolin-1 expression in SMMC-7721 hepatoma cells.** (**A**) HBx expression was examined by western blot (48 hours post-infection). SMMC-7721 cells do not express endogenous HBx, while HBx was effectively produced after HBx transfection. (**B**, **C**) Caveolin-1 mRNA expression in mock- and HBx-transfected cells was examined by real-time PCR. The grey value of the images was measured with Quantity One 4.5. Caveolin-1 in HBx cells was significantly less than that in mock cells (P = 0.022) (examined at 48 hours post-infection). (**D**) Caveolin-1 protein expression in HBx cells, examined by western blotting, was significantly less than that in mock cells.

SMMC-7721 is a hepatoma cell line that is not infected with HBV. We found that HBx was successfully expressed following transfection into SMMC-7721 cells (Figure 2A). Caveolin-1 was significantly decreased at both mRNA and protein levels after Hbx transfection (Figure 2B–D), indicating that HBx downregulated caveolin-1 expression in SMMC-7721 hepatoma cells.

### HBx induces methylation in the promoter region of the caveolin-1 gene in SMMC-7721 cells

Using immunohistochemical staining to detect caveolin-1 in HBV-positive HCC and adjacent normal tissues, we found that the expression of caveolin-1 was decreased significantly in HCC, compared with that in adjacent tissues (Figure 1I). To examine whether there was DNA methylation in the promoter region of the caveolin-1 gene in HBV-infected HCC tissues (Figure 1A), we performed n-MSP analysis of 33 HCC samples. We found that 84.8% (28/33) were positive for methylation, whereas only 15.2% (5/33) did not show methylation (Figure 1B), indicating that methylation in the promoter region of caveolin-1 was prevalent among HBV-infected HCC patients. However, between HCC and adjacent non-tumor tissue in these specimens, we found that the HCC tissue was positive for methylation, but it was negative for methylation in the adjacent non-tumor tissue in the same specimens (Figure 1J). We also examined the methylation status in SMMC-7721 hepatoma cells and found that the caveolin-1 promoter was not methylated in mock-treated cells, whereas HBx-transfected cells were positive for both methylation and non-methylation. This result indicated that methylation in the caveolin-1 promoter region was caused by HBx in SMMC-7721 cells (Figure 1C). To identify CpG methylation sites in the caveolin-1 promoter, we sequenced unmethylated and methylated PCR products. The unmethylated product (Pmd-18 T-Cav-U) corresponded to the −411 ~ −181 segment of the caveolin-1 promoter, whereas the methylated product (Pmd-18 T-Cav-M) corresponded to the – 401 ~ −191 segment of the caveolin-1 promoter and contained 16 CpG sites. We found that there was only one methylated CpG site in unmethylated PCR products amplified from control SMMC-7721 cells, whereas there were 16 methylated CpG sties in methylated PCR products amplified from HBx-transfected SMMC-7721 cells, suggesting that HBx induced methylation of the other 15 CpG sites in this region (Figure 1D). We also found that 5′-Aza-2′-DC treatment could reverse HBx-induced methylation in the caveolin-1 promoter (Figure 1E). Moreover, caveolin-1 was significantly increased at both the mRNA and protein levels after 5′-Aza-2′-DC treatment (Figure 1F–G). Next, a dual-luciferase reporter assay was conducted to examine the transcriptional activity of the caveolin-1 promoter. We found that exogenous HBx could significantly downregulate caveolin-1 promoter activity (P = 0.001), whereas 5′-Aza-2′-DC treatment inhibited such an effect. This result indicated that HBx decreases caveolin-1 expression by DNA methylation-mediated suppression of the caveolin-1 promoter (Figure 1H).

## Discussion

In tumor cells, hypermethylation of CpG islands in the promoter regions of tumor suppressor genes often results in transcriptional silencing. This event often correlates with loss of protein expression, which is widely recognized as a major contributing factor in tumorigenesis and malignancy. Hypermethylation of CpG islands in several tumor suppressor genes, including p53, p16, E-cadherin, Nm23, and MLH1, has been reported in several types of malignant tumors [10-15].

Recent studies revealed that the caveolin-1 promoter region is hypermethylated in lung, breast, cervical and bladder cancers, as well as leukemia [8,9,16,17]. Hirasawa Y et al. reported that 38% of HCC tissues show hypermethylation in the promoter region of the caveolin-1 gene. In their report, hypermethylation was also found in the hepatoma cell line Hep3B that is infected with HBV [18]. In our current study, we found that most (84.8%, 28/33) HBV-infected HCC tissues showed hypermethylation in the promoter region of the caveolin-1 gene, indicating that this epigenetic change is prevalent among patients with HBV-related HCC. As a classic qualitative method for DNA methylation, n-MSP is not accurate enough for evaluating the level of DNA methylation. It would be better to use Methylight assays to show the level of DNA methylation and to correlate it to the level of expression of caveolin-1 and HBx using quantitative assays for expression analysis such as qRT-PCR or Western blotting. However, there was not a sufficient amount of HCC tissues to perform such further experiments. So, the correlation between DNA methylation and the expression of corresponding mRNA and protein would be researched using quantitative assays in future studies.

Many viral infections, including HBV, induce hypermethylation in the promoter regions of many tumor suppressor genes, which results in transcriptional silencing and loss of protein expression. Jung et al. reported that HBx induces hypermethylation in the promoter region of E-Cadherin, which results in its suppression [19]. A negative correlation between HBx and caveolin-1 expression was found in our previous study based on a cohort of patients with HBV-related HCC [6]. This observation suggests that a similar mechanism, in which caveolin-1 is repressed by HBx-mediated DNA methylation, might exist. In fact, we found that transfection of HBx significantly suppressed the transcriptional activity of the caveolin-1 promoter and decreased caveolin-1 expression at the protein level. Next, we investigated the DNA sequence of the caveolin-1 promoter to identify CpG islands. We found that HBx methylated CpG sites within the caveolin-1 promoter. Because 5′-Aza-2′-DC inhibits methylation of CpG islands by inhibiting DNA methyltransferase (DNMT) activity, we used 5′-Aza-2′-DC to treat HBx-transfected SMMC-7721 cells. We found that the promoter region of caveolin-1 was almost completely unmethylated following treatment. Moreover, the suppressive effect of HBx on caveolin-1 expression was also completely abolished, indicating that HBx-mediated DNA methylation is the leading cause of caveolin-1 suppression.

Our previous study found that caveolin-1 expression is significantly decreased in HCC. In addition, caveolin-1 expression inversely correlates with the tumor size, alpha fetal protein level, vascular invasion, tumor number and pathological TNM (pTNM) staging, which implied that the expression of caveolin-1 gradually decreased with HCC progression. MHCC-97 H and MHCC-97 L cells are derived from parental MHCC-97 cells. MHCC-97 H cells are more invasive and malignant, and express less caveolin-1 than MHCC-97 L cells, which is consistent with its possible role as a tumor suppressor [6]. Interestingly, we recently found that overexpression of caveolin-1 decreased tumor invasion and metastasis (unpublished data), supporting the hypothesis that caveolin-1 suppresses tumorigenesis.

## Conclusions

HBV is one of the leading causes of HCC. For the first time, we report that HBx induces hypermethylation of the promoter region of the caveolin-1 gene, suppresses its transcriptional activity, and decreases its expression. Our results indicate that this epigenetic regulation of caveolin-1 by HBx might play a major role in HCC development.

## Abbreviations

HBx: Hepatitis B virus X protein; HBV: Hepatitis B virus; n-MSP: Nested methylation-specific PCR; HCC: Hepatocellular carcinoma.

## Competing interests

The authors declare no conflict of interest.

## Authors’ contributions

JY carried out the genetic studies and participated in drafting the manuscript. QL carried out the immunoassays. KSMa and LC participated in the clinical studies. JHD participated in study design and performed the statistical analyses. XWL conceived the study, participated in its design and coordination and helped to draft the manuscript. All authors read and approved the final manuscript.

## Pre-publication history

The pre-publication history for this paper can be accessed here:

http://www.biomedcentral.com/1471-2407/12/353/prepub
